# Cerebral Small Vessel Disease: Targeting Oxidative Stress as a Novel Therapeutic Strategy?

**DOI:** 10.3389/fphar.2016.00061

**Published:** 2016-03-17

**Authors:** T. Michael De Silva, Alyson A. Miller

**Affiliations:** ^1^Department of Pharmacology, Biomedicine Discovery Institute, Monash UniversityMelbourne, VIC, Australia; ^2^Cerebrovascular and Stroke Laboratory, School of Health and Biomedical Sciences, RMIT UniversityMelbourne, VIC, Australia

**Keywords:** cerebral small vessel disease, oxidative stress and redox regulation, NADPH oxidases (Noxs), brain injury, cognitive impairment, pharmacological strategies

## Abstract

Cerebral small vessel disease (SVD) is a major contributor to stroke, and a leading cause of cognitive impairment and dementia. Despite the devastating effects of cerebral SVD, the pathogenesis of cerebral SVD is still not completely understood. Moreover, there are no specific pharmacological strategies for its prevention or treatment. Cerebral SVD is characterized by marked functional and structural abnormalities of the cerebral microcirculation. The clinical manifestations of these pathological changes include lacunar infarcts, white matter hyperintensities, and cerebral microbleeds. The main purpose of this review is to discuss evidence implicating oxidative stress in the arteriopathy of both non-amyloid and amyloid (cerebral amyloid angiopathy) forms of cerebral SVD and its most important risk factors (hypertension and aging), as well as its contribution to cerebral SVD-related brain injury and cognitive impairment. We also highlight current evidence of the involvement of the NADPH oxidases in the development of oxidative stress, enzymes that are a major source of reactive oxygen species in the cerebral vasculature. Lastly, we discuss potential pharmacological strategies for oxidative stress in cerebral SVD, including some of the historical and emerging NADPH oxidase inhibitors.

## Introduction

Cerebral small vessel disease (SVD) is the term currently used to pathological processes that affect the brain parenchymal circulation (arterioles, capillaries, and veins). Brain parenchymal arterioles are an important site of vascular resistance (Faraci, 2011) and are crucial for maintaining adequate blood flow to the sub-surface brain structures (Bullmore and Sporns, 2012). The brain requires a disproportionate amount of the body’s energy. Despite accounting for only 2% of body mass, up to 20% of cardiac output is devoted to meeting the brain’s energy demands (Attwell et al., 2010). In order to deliver these nutrients effectively and protect the brain from hypoperfusion and ischemic damage, the cerebral vasculature possesses well-developed mechanisms that enable cerebral blood flow (CBF) to remain constant during fluctuations in arterial pressure (autoregulation) and to meet the increased nutrient demands when local brain activity rises (neurovascular coupling; Cipolla, 2009; Jackman and Iadecola, 2014; De Silva and Faraci, in press). Cerebral SVD significantly and chronically impairs the ability of the cerebral vasculature to meet these demands due to a number of structural and functional changes, which ultimately result in brain injury, cognitive decline, and dementia.

Cerebral SVD takes an enormous toll on the individual and is a major burden on the healthcare system. It not only accounts for 20–30% of all strokes (Pantoni, 2010), but significantly increases the risk of future stroke (Vermeer et al., 2007; Debette and Markus, 2010). Furthermore, cerebral SVD is thought to account for about 45% of dementia cases (Gorelick et al., 2011). Cerebral SVD is more prevalent in the elderly. As such, efforts to study and develop treatments for cerebral SVD need to be intensified in order to reduce both the social and economic burden of the disease as our population ages.

In this review, we will discuss the clinical features, pathological changes, and impact of cerebral SVD on the brain and its vasculature. We will briefly discuss the two most common categories of SVD, non-amyloid and amyloid forms. We will then discuss oxidative stress and the potential role it may play in the arteriopathy of cerebral SVD, as well as its role in cerebral SVD-related brain injury and cognitive impairment. Also, we will highlight evidence implicating the NADPH oxidases as key contributors to the development of oxidative stress. Finally, we will discuss the therapeutic agents including anti-oxidant strategies, and their potential use for the treatment of cerebral SVD.

## What is Cerebral Small Vessel Disease?

### Non-Amyloid Cerebral SVD

The clinical manifestations of cerebral SVD vary depending on the specific cause of the disease (i.e., sporadic vs. monogenic origin) as well as the brain region(s) affected. Individuals may present with sudden onset stroke symptoms, changes in cognitive function, progressive cognitive deterioration, dementia, depression and physical disability (Chabriat et al., 1995; van der Flier et al., 2005; Inzitari et al., 2009; de Laat et al., 2011). It is thought that parenchymal arterioles, capillaries and venules are the preferential target of cerebral SVD. As these vessels are difficult to directly assess in patients *in vivo* (Wardlaw et al., 2001), diagnosis relies on a range of clinical, cognitive, neuroimaging, and neuropathological tests. The majority of cases of cerebral SVD are sporadic, with aging and hypertension thought to be the most important risk factors. A number of hereditary forms of cerebral SVD have also been identified (See Haffner et al., 2015 for discussion). The difficulty in studying small cerebral vessels has likely contributed to the lack of understanding of the disease and absence of any specific pharmacological strategies for its treatment.

Cerebral SVD induces a number of pathological changes to the vasculature. In small arterioles, this may include marked vascular muscle dysfunction, lipohyalinosis, vascular remodeling, and deposition of fibrotic material. Basement membranes can also become thickened and perivascular spaces enlarged. There may also be disruption of the blood-brain barrier (BBB) leading to edema (Taheri et al., 2011). Venous structure is also affected with collagen being deposited in the walls of venules (venous collagenosis; Moody et al., 1995). These diverse changes to the cerebral microvasculature result in reduced CBF (resulting in chronic hypoperfusion) and a loss of adaptive responses (e.g., autoregulation and neurovascular coupling). As a result the ability to adequately supply the brain with the required nutrients is significantly impaired, resulting the profound tissue damage.

Diagnosis of cerebral SVD relies in large part on neuroimaging findings. Wardlaw et al. (2013) has described in detail the changes that occur in the brain during sporadic cerebral SVD and the use of imaging techniques to detect these changes. The features that can be detected using imaging techniques such as magnetic resonance imaging (MRI) include lacunar infarcts/hemorrhages, white matter hyperintensities (WMH), dilated perivascular spaces, and brain atrophy (Doubal et al., 2010; Rost et al., 2010; Jokinen et al., 2011; Aribisala et al., 2013; Potter et al., 2015). Use of more advanced MRI techniques reveals further brain injury including brain edema, and further alterations to white matter (Bastin et al., 2009; Maclullich et al., 2009). One of the difficulties in diagnosing cerebral SVD is that these markers are not specific for SVD alone. For example, the presence of WMH is not restricted to cerebral SVD, and lacunar infarcts may occur due to an embolism (Jackson et al., 2010; Potter et al., 2012). Therefore, clinicians rely on the presence of a number of these features for proper diagnosis of the disease.

The etiology of cerebral SVD is incompletely understood. Cardiovascular risk factors such as hypertension and aging are thought to be important contributors to late life dementia (Hall et al., 2005; Kivipelto et al., 2006; Gottesman et al., 2014). Such risk factors are likely to worsen disease progression via deleterious effects on both the structure and functioning of cerebral blood vessels. Another thought provoking hypothesis is that failure of the BBB, leading to extravasation of toxic plasma components (Silberberg et al., 1984), may be an important factor in cerebral SVD. BBB disruption is linked with brain injury caused by a number of neurological conditions including stroke, multiple sclerosis, and Alzheimer’s disease. Wardlaw et al. (2013) proposed that endothelial cell failure during cerebral SVD would lead to extravasation of toxic plasma components resulting in localized damage to both the blood vessel and brain parenchyma. Additional research is needed to fully define the role of BBB failure in the pathogenesis of cerebral SVD.

Interestingly, while cerebral SVD primarily affects the microvasculature, it has been suggested that larger arteries may also contribute to the disease process (Xu, 2014). Specifically, lacunar strokes may occur as a result of atheroma or cardiac embolism (Wardlaw et al., 2013). Furthermore, increased arterial stiffness has been shown to be associated with an increased white matter lesion burden (Poels et al., 2012). Therefore, while the microvasculature is the primary target of SVD, the contribution of larger arteries should not be immediately discounted.

### Amyloid Cerebral SVD

Cerebral amyloid angiopathy (CAA) is a common form of cerebral SVD and refers to the deposition of amyloid β-peptide (Aβ) in the walls of cerebral leptomeningeal and parenchymal arteries, and arterioles. CAA is a frequent observation in the elderly, appearing in ∼10–30% of brain autopsies and 50–80% of people with dementia (Jellinger and Attems, 2010). CAA is most commonly recognized as a cause of hemorrhagic stroke, however, evidence indicates that CAA is also an important contributor to cognitive impairment and dementia (Gorelick et al., 2011). Moreover, CAA is associated with a high prevalence of WMH and cortical microinfarcts, which are assumed to be of ischemic origin (Haglund and Englund, 2002; van Horssen et al., 2005; De Reuck et al., 2011). Mechanisms linking CAA and brain injury, whether that be hemorrhagic or ischemic in nature, are not completely understood, however, they are likely to involve both structural and functional abnormalities of the cerebral vasculature. For example, in advanced cases, CAA can cause structural changes such as concentric splitting, loss of smooth muscle cells, and fibrinoid necrosis, which may increase the propensity for vessel rupture and thus hemorrhage. Clinical and experimental evidence indicates that deposition of Aβ in the vessel wall can also cause functional abnormalities (e.g., impaired vasodilator responses and autoregulation; Smith et al., 2008; Park et al., 2014a), which may impair brain perfusion, reduce vascular reserve, and ultimately increase the propensity to ischemic injury. In support of this notion, mice with advanced CAA develop larger infarcts and greater deficits in CBF after transient ischemic stroke (Milner et al., 2014). Evidence, albeit largely from experimental studies, indicates that Aβ may cause cerebral vascular dysfunction even in the absence of vascular deposition (Niwa et al., 2002; Park et al., 2005). Thus, disruptions in the functioning of cerebral vessels by soluble Aβ might be an early event in CAA.

Although CAA is usually observed in large cerebral vessels and arterioles, Aβ depositions can also occur in capillaries (capillary CAA; Attems, 2005). In light of the deleterious effects of Aβ on large vessels and arterioles, it is not surprising therefore to find that Aβ accumulation in capillaries affects BBB integrity. Indeed, BBB leakage and a loss of tight junction proteins occur in patients with CAA (Carrano et al., 2011; Hartz et al., 2012). Experimental studies also provide evidence that Aβ can promote BBB disruption. For example, exogenous Aβ triggers tight junction disruption, cell death, and hyperpermeability of cultured rodent and human brain endothelial cells (Marco and Skaper, 2006; Gonzalez-Velasquez et al., 2008; Carrano et al., 2011; Hartz et al., 2012). Also, microvessels of mice with CAA display increased BBB permeability (Lee et al., 2005; Hartz et al., 2012; Kruyer et al., 2015). In addition to its effects on endothelial cells of the BBB, Aβ might have deleterious effects on other cells of the neurovascular unit. For example, exogenous Aβ induces death of cultured human brain pericytes (Verbeek et al., 1997; Bruinsma et al., 2010), and pericyte loss is evident in mice with advanced CAA (Park et al., 2014a).

## Reactive Oxygen Species and Cerebral Vascular Function

### ROS Generation in the Vasculature

Reactive oxygen species (ROS) are a group of oxygen-derived molecules generated by all mammalian cells. The conversion of molecular oxygen to superoxide by oxidase enzymes is the first step in the generation of all ROS. Superoxide can be converted either enzymatically [by superoxide dismutases (SOD)] or spontaneously to the non-radical hydrogen peroxide. Furthermore, hydrogen peroxide can be generated directly by some enzymes (e.g., the NADPH oxidases; Dikalov et al., 2008). Hydrogen peroxide is chemically more stable than superoxide and can easily cross cellular membranes. As such, hydrogen peroxide may be the most important ROS in terms of physiological cell signaling. Glutathione peroxidase (Gpx) or catalase enzymatically metabolize hydrogen peroxide to water and oxygen. During physiological conditions, the production and metabolism of ROS by anti-oxidants is tightly regulated in order to prevent cellular damage. Under these conditions, ROS may serve as important signaling molecules for the regulation of vascular tone (Miller et al., 2005, 2006; Paravicini et al., 2006). Numerous stimuli can, however, disrupt the oxidant/anti-oxidant balance and the ultimate effect is one where oxidants overwhelm anti-oxidants resulting in oxidative stress. Compelling evidence indicates that oxidative stress underlies cerebral vascular dysfunction associated with a number of vascular-related diseases (see Oxidative stress and cerebral vascular dysfunction).

### NADPH Oxidases: A Key Source of ROS in the Cerebral Vasculature

Cerebral arteries express a number of enzymes that are potential sources of ROS including cyclooxygenase (COX), mitochondria, and the NADPH oxidases. COX is an important source of vascular ROS in cerebral arteries (Kontos et al., 1984, 1990; Didion et al., 2001). COX generates superoxide during the conversion of arachidonic acid to prostaglandin H_2_, which is the precursor for many vasoactive prostanoids. Therefore, the ROS generated by COX is a by-product of its normal function. Mitochondria generate ROS during oxidative phosphorylation and are another important source of ROS in the vasculature (Busija and Katakam, 2014). Cerebral endothelial cells contain a relatively high density of mitochondria (Oldendorf et al., 1977), which may in part reflect the high metabolic demands of maintaining the BBB. As with COX, mitochondrial-derived ROS are by-products of its normal function. It has been suggested that endothelial NO synthase (eNOS) can switch from generating NO to superoxide when it is “uncoupled.” Studies have shown that reduced bioavailability of tetrahydrobiopterin, a requisite cofactor for eNOS activity, results in eNOS-dependent superoxide production, rather than NO (Santhanam et al., 2012, 2014).

As is discussed above, COX, mitochondria and eNOS generate ROS as a by-product of their normal enzymatic function or in a dysfunctional state. In contrast, the NADPH oxidases are the only known family of enzymes that generate ROS as their primary function (Drummond et al., 2011). The NADPH oxidase is a heteromultimeric enzyme complex with up to six separate subunits. Nox2-NADPH oxidase or gp91phox was the first NADPH oxidase to be discovered in neutrophils, where this enzyme plays a central role in immunological host defense (Babior et al., 2002). Since this discovery, a number of other NADPH oxidase isoforms have been identified, namely Nox1-, Nox3-, Nox4-, and Nox5-NADPH oxidases. The structure and functioning of the Nox isoforms have been comprehensively reviewed (Selemidis et al., 2008; Drummond et al., 2011; Lassegue et al., 2012) and thus will not be discussed here. With respect to the cerebral circulation, Nox1, Nox2, and Nox4 catalytic subunits, as well as cytoplasmic subunits (e.g., p47phox, p67phox), are expressed at the mRNA and protein level (Kazama et al., 2004; Paravicini et al., 2004; Ago et al., 2005; Miller et al., 2007). Notably, Nox4 appears to be the most abundant isoform in cerebral arteries (during health) followed by Nox1 and finally Nox2 (Ago et al., 2005). Substantial evidence indicates that the NADPH oxidases are functionally active in cerebral arteries, and are a major source of ROS in cerebral arteries during health (Didion and Faraci, 2002; Park et al., 2004a; Miller et al., 2005). Moreover, compelling evidence indicates that excess production of superoxide by these enzymes underlies the oxidative stress and cerebral vascular dysfunction that is associated a number of vascular-related diseases (e.g., hypertension, diabetes, hypercholesterolemia) and also advanced age (Mayhan et al., 1990; Girouard et al., 2006; Kitayama et al., 2007; Miller et al., 2010).

### Roles of ROS and the NADPH Oxidases in Regulating Cerebral Vascular Function

In blood vessels from healthy animals (e.g., mouse, rat, pig, and rabbit), NADPH oxidase activity and ROS generation are significantly higher in cerebral arteries compared with systemic arteries (Miller et al., 2005, 2009). Moreover, this is associated with greater expression levels of the Nox2- and Nox4-NADPH oxidase isoforms (Miller et al., 2005, 2009). This may reflect an important role for ROS-dependent signaling in cerebral arteries. Indeed, ROS have been shown to be important vasoactive molecules in cerebral arteries from healthy animals. However, this potentially means that a smaller increase in ROS levels is needed before antioxidant defense mechanisms are overcome and oxidative stress ensues. The majority of studies examining the effects of exogenously applied or endogenously generated ROS infer that they are cerebral vasodilators during health. For example, application of NADPH (substrate for the NADPH oxidases) to cerebral vessels *in vitro* and *in vivo* elicits vasodilation (Didion and Faraci, 2002; Park et al., 2004a; Miller et al., 2005) that is dependent on hydrogen peroxide (Miller et al., 2005). Moreover, NADPH-oxidase–derived ROS partly mediate flow-dependent responses of rat cerebral arteries *in vivo* (Paravicini et al., 2006). As such, it has been proposed that ROS derived from NADPH oxidases may play an important physiological role in regulating cerebral vascular tone and thus potentially brain perfusion (Miller et al., 2006).

## Oxidative Stress and Cerebral Vascular Dysfunction

Oxidative stress is characterized by an imbalance between the production and metabolism of oxidants in favor of a pro-oxidant cellular environment. Excess oxidant levels can then overwhelm the endogenous anti-oxidant systems. Oxidative stress can result from overproduction of oxidants [in the form of ROS and reactive nitrogen species (RNS)] by enzymes such as the NADPH oxidases or from diminished anti-oxidant enzyme activity or anti-oxidant levels (e.g., SODs, Gpx, and catalase).

Oxidative stress has major implications for cerebral vascular function. Substantial evidence implicates oxidative stress as a key contributor to the pathogenesis of many diseases that affect the brain and its circulation. Indeed, as discussed in the following sections, oxidative stress, driven primarily by the NADPH oxidases may play an important role in the cerebral vascular dysfunction associated with cerebral SVD risk factors and CAA. It is well documented that oxidative stress can impair cerebral vascular function via the disruption of endothelium-dependent NO signaling (Girouard et al., 2006, 2007; Mayhan et al., 2008; Miller et al., 2010). Indeed, the reaction of superoxide with NO reduces NO bioavailability and thus diminishes its potent vasodilator, anti-proliferative, and anti-inflammatory properties. Importantly, this reaction also generates the RNS (ONOO^-^). ONOO^-^ is a powerful oxidant and can further accentuate vascular dysfunction by causing damage to proteins and DNA, and by impairing vascular responses to NO (Xie et al., 2005). Although evidence points toward hydrogen peroxide being a vasodilator ROS in the cerebral circulation, high concentrations may lead to apoptosis of cerebral vascular cells (Li et al., 2003). Moreover, this non-radical may impair vascular function through its conversion into hydroxyl (OH^.^) in the metal-catalyzed Haber–Weiss or Fenton reaction (Xie et al., 2005).

Another critically important consequence of reduced NO bioavailability during disease is the loss of modulation of Rho kinase activity. NO has been shown to modulate Rho kinase activity in cerebral microvessels, such that inhibition of NO synthase activity increases the influence of Rho kinase on vascular tone (Didion et al., 2005). In addition, ROS may be able to directly increase Rho kinase signaling (Jernigan et al., 2008; Aghajanian et al., 2009). In turn, Rho kinase can influence both eNOS expression (via effects on eNOS mRNA stability) and activity (Laufs and Liao, 1998; Faraco et al., 2013). Thus, the loss of NO during disease can start a viscous cycle, increased Rho kinase activity leading to decreased NO synthase-derived NO, which may further increase Rho kinase activity. Other mechanisms of ROS-mediated vascular damage have been described during non-amyloid and amyloid cerebral SVD disease states, including activation of poly(ADP)-ribose polymerase (PARP) and transient receptor potential melestatin-2 (TRPM2) channel activation. Our current understanding of these mechanisms is discussed below.

## Does Oxidative Stress Contribute to Cerebral Vascular Dysfunction in Cerebral SVD?

### Non-amyloid Cerebral SVD and its Risk Factors

Sporadic SVD is difficult to model and study experimentally. As such, research efforts to elucidate the arteriopathy of cerebral SVD have largely focused on the use of experimental models of its risk factors. Many of the key features of cerebral SVD arteriopathy have been described in such models. However, there is a lack of evidence supporting a causal link between these vascular abnormalities and, brain injury and cognitive impairment. This latter point notwithstanding, experimental models of cerebral SVD risk factors have revealed that oxidative stress might be a common underlying mechanism responsible for cerebral vascular dysfunction.

As mentioned, hypertension and aging are thought to be the most important risk factors for cerebral SVD. The harmful effects of hypertension on the cerebral vasculature have been extensively studied and described. For example, some of the key features of cerebral SVD have been described in rodent models of hypertension including reductions in CBF, impaired vasodilator capacity (e.g., NO-dependent responses, neurovascular coupling, and autoregulation), inward remodeling, and BBB disruption (Didion et al., 2000; Kim-Mitsuyama et al., 2005; Faraci et al., 2006; Girouard et al., 2006, 2007; Henning et al., 2010). Angiotensin II, the main effector peptide of the renin-angiotensin system, appears to play an important role in these vascular abnormalities (De Silva and Faraci, 2012). Moreover, compelling evidence indicates that oxidative stress, driven primarily by the NADPH oxidases, mediates many of the deleterious effects of angiotensin II on the cerebral vasculature. For example, angiotensin II acutely and chronically, increases superoxide production by Nox2-NADPH oxidase in rodent cerebral vessels (Girouard et al., 2006, 2007; De Silva et al., 2009; Jackman et al., 2009b; De Silva and Faraci, 2012). Also, functional impairments of mouse cerebral arterioles following angiotensin II treatment are prevented by co-treatment with either the ROS scavenger MnTBAP or the Nox2-NADPH oxidase peptide inhibitor gp91ds-tat (Girouard et al., 2006). Furthermore, studies of mice genetically deficient of Nox2 also suggest a role for Nox2-NAPDH oxidase in the functional abnormalities caused by angiotensin II (Girouard and Iadecola, 2006; Girouard et al., 2006, 2007; Chrissobolis et al., 2012). More recently, studies have shown that angiotensin II can also activate and increase superoxide production in cerebral vessels by activating Nox1-NADPH oxidase (Jackman et al., 2009a). However, this isoform appears to only play a minor role in the vascular abnormalities caused by angiotensin II (Chrissobolis et al., 2012).

Several studies have shown that oxidative stress and the NADPH oxidases also mediate the deleterious effects of hypertension/angiotensin II on the microcirculation. For example, scavenging of superoxide by tempol or the NADPH oxidase inhibitor apocynin reduce BBB disruption in spontaneously hypertensive stroke prone rats (SHRSP) rats treated with angiotensin II (Kim-Mitsuyama et al., 2005). Similarly, tempol attenuates leukocyte/endothelial interactions and BBB disruption in mice treated with angiotensin II (Zhang et al., 2010). BBB disruption, tight junction protein loss, and oxidative stress have also been reported in other models of hypertension such as Dahl salt-sensitive rats (Kalani et al., 2016). At present, the downstream mechanisms responsible for the ROS-mediated BBB dysfunction have not been identified. However, studies of cerebral ischemia and reperfusion implicate Rho kinase activation as a key mechanism underpinning ROS-induced BBB disruption (Kahles et al., 2007). The potential involvement of Rho kinase in BBB dysfunction during hypertension and cerebral SVD warrants further investigation.

Oxidative stress has been shown to play a critical role in mediating changes to cerebral vascular structure. Changes in arteriolar structure have profound effects on CBF. Inward remodeling results in a reorganization of the vessel wall such that there is a reduction in lumen diameter that does not result from changes in vessel reactivity or mechanics (Faraci, 2011). Also, inward remodeling reduces lumen diameter even when vessels are fully dilated (Baumbach and Heistad, 1989; Baumbach et al., 2003). Nox2-deficient mice are protected from the cerebral vascular hypertrophy and inward remodeling induced by angiotensin II (Chan and Baumbach, 2013a,b), implicating a role for Nox2-NADPH oxidase-derived ROS in these structural abnormalities. The precise signaling cascade through which ROS mediates these changes is yet to be elucidated. Recent studies indicate that a signaling cascade involving caveolin-1-containing microdomains, epidermal growth factor receptor (EGFR), c-Src and Akt is required for the hypertrophic response to angiotensin II in cerebral arterioles (Chan et al., 2015; Umesalma et al., 2016). Moreover, increased expression and activation of matrix metalloproteinase-9 (MMP-9) appears to be crucial for the cerebral vascular inward remodeling (Umesalma et al., 2016). Whether ROS activate these signaling cascades to mediate cerebral vascular remodeling remains to be tested.

Another important structural change that is associated with cerebral SVD is vessel rarefaction (particularly in the white matter; Brown et al., 2007), which is the loss of collateral vessel number and a decrease in the diameter of remaining vessels (De Silva and Faraci, in press). Rarefaction was recently shown to occur in cerebral pial arterioles and capillaries during hypertension, aging, diabetes, hypercholesterolemia, and metabolic syndrome (Faber et al., 2011; Moore et al., 2015). Furthermore, the impact of hypertension on collateral vessels was worsened by the severity and duration of the hypertension (Moore et al., 2015). While direct evidence for a role for ROS and NADPH oxidase is currently lacking, the fact that oxidative stress has been shown to be present in hypertension, albeit with different end points, raises the possibility that ROS and NADPH oxidases may play a role in rarefaction. Consistent with this, chronic blockade of angiotensin II type 1 receptors (AT_1_) and angiotensin-converting enzyme (ACE) reverses brain functional capillary rarefaction and lowers brain oxidative stress in diabetic hypertensive rats (Estato et al., 2013). Recent evidence highlights the importance of pericytes in regulation microcirculation, brain capillary perfusion, cerebral vascular responses, and BBB integrity. Indeed, pericyte deficient mice display marked reductions in microcirculation and BBB disruption (Bell et al., 2010). To the best of our knowledge, it is unclear whether hypertension influences pericyte function and number, and whether oxidative stress contributes to any such effect. However, given pericytes express Nox-NADPH oxidases and generate ROS in response to angiotensin II (Kuroda et al., 2014), it is conceivable that oxidative stress may have deleterious effects on pericytes during hypertension.

Similar to hypertension, it is well documented that diverse vascular changes occur with aging. In the human and rodent cerebral circulation, aging produces functional abnormalities including reduced resting CBF and impaired endothelium-NO-dependent responses and neurovascular coupling (Hatake et al., 1990; Mayhan et al., 1990, 2008; Park et al., 2007; Modrick et al., 2009, 2012; Pena Silva et al., 2012). Also, studies of aged rodents have shown that cerebral arterioles undergo structural changes such as atrophy, reduced distensibility, and a reduction in elastin and smooth muscle (Hajdu et al., 1990). Similarly, in humans, aging increases wall thickness and reduces distensibility of large cerebral arteries such as the basilar and vertebral arteries (Nagasawa et al., 1979). Studies suggest a major role for oxidative stress and the NADPH oxidases in age-related cerebral vascular abnormalities. For example, acute scavenging of superoxide by tempol or MnTBAP improves NO-dependent vasodilator responses and neurovascular coupling in aged mice (Mayhan et al., 2008; Modrick et al., 2009). Inhibition of NADPH oxidases with apocynin, diphenyleniodonium (DPI) or gp91ds-tat, also improves cerebral vascular function in aged mice (Mayhan et al., 2008). Also, age-related cerebral vascular abnormalities are not found in Nox2-deficient mice (Park et al., 2007). The renin-angiotensin system may contribute to the vascular changes in aging. Indeed, levels of angiotensin II, ACE, and AT_1_ receptors are increased in peripheral vessels of aged monkeys and old humans (Wang et al., 2003, 2007). Moreover, genetic deletion of AT_1_ attenuates cerebral vascular dysfunction in aged mice (Modrick et al., 2009). In addition to oxidative inactivation of NO, studies have shown that activation of the DNA repair enzyme poly(ADP-ribose) polymerase (PARP) by ROS contributes to cerebrovascular endothelial dysfunction during aging (Modrick et al., 2009). To the best of our knowledge, no study has examined whether oxidative stress and the NADPH oxidases contribute to age-related structural abnormalities of cerebral vessels. Moreover, it is unknown whether oxidative stress contributes to age-related rarefaction of arterioles and capillaries, and pericyte loss (Bell et al., 2010; Faber et al., 2011).

Studies of experimental models of other cerebral SVD risk factors such as hypercholesterolemia (Kitayama et al., 2007; Miller et al., 2010), diabetes (Mayhan et al., 1991, 2006; Didion et al., 2005, 2007; Arrick et al., 2008; Ergul et al., 2009), obesity (Lynch et al., 2013), and homocysteinaemia (Dayal et al., 2004, 2005; Devlin et al., 2004), indicate that all are associated with functional abnormalities and oxidative stress. For example, genetic deletion of Nox2 in hypercholesterolemic apolipoprotein E-deficient mice prevents elevations in ROS production and impaired vasodilator capacity of cerebral vessels (Miller et al., 2010). Similarly, Nox2-deficient mice are protected against obesity-induced dysfunction of cerebral arterioles (Lynch et al., 2013). Moreover, activation of PARP (Arrick et al., 2007), Rho kinase (Didion et al., 2005), and AT_1_R (Arrick et al., 2008) have also been shown to be involved in cerebral microvascular dysfunction. Thus, as in models of hypertension and aging, the generation of ROS by NADPH oxidases and the activation of key signaling pathways such as PARP and Rho kinase might be a common underlying mechanism of cerebral vascular dysfunction caused by many of the known risk factors for cerebral SVD.

### Amyloid Cerebral SVD

The mechanisms underlying CAA-induced vascular abnormalities remain to be fully elucidated. In the scenario where Aβ deposition occurs, it could involve alterations in vasomotor apparatus, including the loss of smooth muscle cells due to the toxic properties of Aβ (Ruzali et al., 2013). Moreover, Aβ may also present a mechanical obstacle to vessel dilatation, or by interfering with intracellular signaling pathways (Christie et al., 2001). Notably, oxidative stress is evident in cerebral vessels of aged APP mice with CAA (Hamel et al., 2008; Park et al., 2008, 2014b). Similarly, Garcia-Alloza et al. (2009) found a strong correlation between CAA-positive cerebral vessels and oxidative stress in two APP transgenic mouse models that develop CAA (APPswe/PS1dE9 and Tg2576). In addition, emerging evidence suggests that oxidative stress may play an important role in mediating the deleterious effects of Aβ/CAA on the cerebral vasculature. For example, a recent study by Han et al. (2015) showed that chronic treatment with the ROS scavenger tempol reduces oxidative stress and improves vasodilator responses of cerebral arterioles in aged APP transgenic mice with CAA. Also, they provided evidence that oxidative stress may also contribute to CAA-related microhemorrhage (Han et al., 2015). Park et al. (2014a) also demonstrated that oxidative stress contributes to vascular dysfunction in APP transgenic mice, however, they also showed that with advancing age, structural alterations (e.g., smooth muscle fragmentation and pericyte loss) also play a role. Genetic deletion or inhibition of Nox2 improves the functioning of cerebral vessels of aged APP mice, which typically exhibit CAA (Park et al., 2007, 2008). Similarly, the purported NADPH oxidase inhibitor apocynin, or tempol, reduce oxidative stress and cerebral vascular abnormalities in aged APP mice with CAA (Han et al., 2015). Thus, the NADPH oxidases, in particular the Nox2 isoform, might be a key driver of oxidative stress-induced vascular dysfunction in CAA, however, there is a paucity of studies examining the potential roles of other NADPH oxidase isoforms and/or other ROS enzymatic sources. Although the precise mechanism through which Aβ activates the NADPH oxidases remains to be fully determined, recent evidence implicates a role for CD36, which is an important innate immunity receptor that binds Aβ and is expressed on endothelial cells (Park et al., 2011, 2013).

As discussed, it is well established that superoxide can impair cerebral vascular function by inactivating vasoprotective NO. Moreover, it is well documented that the reaction between superoxide and NO also generates ONOO^-^. In addition to oxidative stress, studies have also found evidence that nitrosative damage may also contribute to the vascular abnormalities in CAA (Park et al., 2014b). Moreover, the molecular mechanism by which ONOO^-^ causes cerebral vascular dysfunction in response to Aβ has been described. Specifically, Park et al. (2014b) showed that Aβ-induced nitrosative stress caused DNA damage, resulting in PARP activation and ultimately an increase in intracellular ADP ribose in cerebral arterioles. This in turn activates TRPM2 channels, leading to a large increase in intracellular calcium and ultimately endothelial dysfunction. Notably, evidence suggests that ROS may also be able to directly activate TRPM2 channels, bypassing the need for PARP activation (Hecquet et al., 2008). As discussed above, PARP has been identified as a downstream target of ROS and mediator of endothelial dysfunction during hypertension, diabetes, and aging. Thus, both amyloid and non-amyloid forms of cerebral SVD appear to converge on similar molecular pathways. Therefore, targeting these pathways would potentially be beneficial in many forms of cerebral SVD.

In the case of soluble Aβ, evidence also implicates oxidative and nitrosative stress as a key mechanism. Cerebral arterioles of young APP transgenic mice, which have increased levels of endogenous Aβ but no CAA, exhibit signs of oxidative stress and nitrosative damage (Park et al., 2004b, 2008). Moreover, genetic overexpression of SOD or treatment with ROS scavengers improves cerebral vascular function in these mice (Iadecola et al., 1999; Nicolakakis et al., 2008; Park et al., 2008). Several lines of evidence indicate that a key mechanism through which soluble Aβ produces oxidative stress and vascular dysfunction involves the activation of Nox2-NADPH oxidase (Park et al., 2005; Hamel et al., 2008). However, future research is needed to investigate whether other NADPH oxidase isoforms also contribute.

Our understanding of the mechanisms underpinning the deleterious effects of Aβ on the microcirculation lags behind that of large vessels and arterioles. Some evidence does, however, support the notion that oxidative stress is common contributing mechanism throughout the cerebral vascular tree. For example, Aβ increases Nox-dependent H_2_O_2_ formation by human microvascular endothelial cells (*in vitro* BBB model) and decreases mRNA levels of tight junctions proteins (Carrano et al., 2011). Genetic deletion of CD36 in APP transgenic mice attenuates damage to smooth muscle cells and pericytes, increases pericyte numbers and improves morphology (Park et al., 2013). Thus, ROS may contribute to pericyte loss in CAA, which in turn is likely to further accentuate BBB disruption. Consistent with this, the aforementioned study found that expression of the tight junction protein ZO-1 is preserved in CD36 deficient APP mice (Park et al., 2013). Interestingly, post mortem studies have shown that the expression of tight junction proteins is diminished in Aβ-laden capillaries, which are surrounded by Nox2-positive activated microglia (Carrano et al., 2011, 2012). Thus, microglial Nox2-NADPH oxidase may be an important source of ROS in the microcirculation.

The role of oxidative stress in the process of Aβ deposition in the vascular wall is less clear. Heterozygous knockdown of SOD2 in APP transgenic mice increases the deposition of Aβ in the cerebral vasculature (Esposito et al., 2006), suggesting a role for mitochondrial ROS in vascular Aβ burden. A more recent study showed that tempol or apocynin markedly reduce CAA formation in aged APP mice (Han et al., 2015), implicating ROS and potentially the NADPH oxidases in vascular amyloid pathogenesis. Several studies have, however, shown no beneficial effect of ‘anti-oxidant’ strategies (e.g., the non-specific ROS scavengers, phenyl-*N*-ter-butyl nitrone; curcumin; and pomegranate juice) on CAA formation and burden (Hartman et al., 2006; Garcia-Alloza et al., 2011), which may relate to issues of potency and specificity of these ROS scavengers.

## Contribution of Oxidative Stress in Cerebral SVD-Related Brain Injury and Cognitive Impairment

Various experimental models have been employed to study the brain lesions and cognitive impairment that typically manifest in sporadic cerebral SVD. These include chronically hypertensive animals (e.g., SHRSP rats), chronic hypoperfusion models (e.g., carotid occlusion or stenosis models), and models of focal or global ischemia induced by surgical or pharmacological occlusion of a cerebral artery [e.g., monofilament or endothelin-1 (ET-1) models]. However, unfortunately no model exhibits all of the key features of human cerebral SVD (Hainsworth and Markus, 2008). This latter point notwithstanding, compelling evidence from studies using these animal models implicate oxidative stress as a key mediator of ischemic brain injury (Dong et al., 2011; Ueno et al., 2015). Moreover, evidence suggests that the NADPH oxidases, expressed in various cell types in the brain, are major sources of this ROS-mediated ischemic injury.

Walder et al. (1997) were the first to implicate Nox2-NADPH oxidase as a key mediator of ischemic brain injury by showing that Nox2-deficient mice have less brain injury after focal ischemia. Numerous subsequent studies have confirmed this finding in focal and global models (Chen et al., 2009; Jackman et al., 2009b; Brait et al., 2010; De Silva et al., 2011; Yoshioka et al., 2011), and have also shown a role for Nox2-NADPH oxidase in ischemia-induced BBB disruption, and dysfunction of larger pial arteries upstream of the BBB (Kahles et al., 2007; De Silva et al., 2011). Studies using mice deficient of Nox4 also suggest a role for Nox4-NADPH oxidase in ischemic brain injury. Indeed, Nox4 deficient mice develop less brain injury after focal ischemia as a result of reduced oxidative stress, neuronal apoptosis, and BBB disruption (Kleinschnitz et al., 2010). The role of Nox1-NADPH oxidase is less clear. Some studies have found no protection in Nox1-deficient mice after focal ischemia (Jackman et al., 2009a; Kleinschnitz et al., 2010), whereas others have shown that knockdown of Nox1 (Choi et al., 2014, 2015) reduces neuronal cell death in rat models of focal or chronic hypoperfusion.

Pharmacological evidence also implicates the NADPH oxidases in ischemic-related brain injury. Indeed, numerous studies have shown that apocynin provides neuroprotection in focal, global and chronic hypoperfusion models (Tang et al., 2008; Chen et al., 2009; Jackman et al., 2009b; Choi et al., 2014; Zhang et al., 2015), protects against BBB disruption (Kahles et al., 2007; Tang et al., 2008) and hemorrhage (Tang et al., 2008), and attenuates enhanced ROS production and neuronal apoptosis in stroke-prone spontaneously hypertensive rats (Yamamoto et al., 2008). Moreover, some studies using Nox2 deficient mice imply that the protection afforded by apocynin involves inhibition of Nox2-NADPH oxidase (Kahles et al., 2007; Chen et al., 2009; Jackman et al., 2009b). Notably, two studies have shown that apocynin has a narrow therapeutic window (Tang et al., 2008; Jackman et al., 2009b) and thus may represent a challenge for therapeutic development. Recently, intrathecal administration of the NADPH oxidase inhibitor VAS2870 was also shown to reduce brain injury after focal ischemia (Kleinschnitz et al., 2010). Moreover, the administration of this inhibitor to Nox4-deficient mice (who are protected themselves) conferred no further benefit, implying that VAS2870 protects against ischemia via inhibition of Nox4-NADPH oxidase (Kleinschnitz et al., 2010).

Experimental studies using natural anti-oxidants also implicate oxidative stress as a contributor in the cognitive impairment associated with cerebral SVD. For example, the polyphenol anti-oxidant, catechin (from green tea), improves learning and memory in rats after chronic cerebral hypoperfusion, an effect that was associated reduced lipid peroxidation and oxidative DNA damage (Xu et al., 2010). L-carnitine also improves learning and memory in rats after chronic cerebral hypoperfusion (Ueno et al., 2015). Moreover, these improvements were associated with reduced lipid peroxidation and oxidative DNA damage, as well as enhanced oligodendrocyte marker expression and myelin sheath thickness indicative of an improvement in white matter lesions (Ueno et al., 2015). Another study found that high doses of Vitamin E improve learning and memory, and reduce markers of oxidative stress (malondialdehyde levels) and cellular injury (lactate dehydrogenase) in stroke-prone hypertensive rats (Murad et al., 2014). As with ischemic-related injury, the NADPH oxidases might contribute to oxidative stress-induced cognitive impairment. For example, apocynin reduces oxidative stress and improves learning and memory in rat or mouse models of chronic hypoperfusion (Shen et al., 2011; Choi et al., 2014; Toyama et al., 2014). Moreover, inhibition of Rac1, which is a small GTPase essential for the assembly and activation of the NADPH oxidases, reduces oxidative stress and improves memory in rats after induction of transient chronic hypoperfusion (Raz et al., 2010). Lastly, Choi et al. (2014) showed that knockdown of Nox1 reduces ROS generation, oxidative DNA damage, and cognitive impairment, implicating a role for the Nox1-NADPH oxidase isoform.

## Pharmacological Strategies for Cerebral SVD

Despite evidence that it has devastating effects on the brain, there are at present no specific pharmacological strategies to prevent or treat cerebral SVD. Given there are a number of well-known risk factors for cerebral SVD one would be predict that prevention (primary and secondary) is a realistic possibility. The benefits for lowering blood pressure with anti-hypertensives are apparent in reducing both first and recurrent stroke. Indeed, in the Systolic Hypertension in the Elderly Program (SHEP), lowering blood pressure with anti-hypertensives reduced the incidence of both hemorrhagic and ischemic stroke, including lacunar (Perry et al., 2000). Also, anti-hypertensive therapy is beneficial for the secondary prevention of subcortical strokes in patients with lacunar infarction (Benavente et al., 2012), and reduces intracerebral hemorrhage (Benavente et al., 2013). Whether anti-hypertensives slow the progression of WMH is less clear. For example, the Perindopril Protection against Recurrent Stroke Study showed that an intensive blood pressure-lowering regime might slow the progression of WMH progression in stroke patients (Dufouil et al., 2005), whereas other trials have noted little or no effect (Bath and Wardlaw, 2015).

Several studies have also demonstrated that statins reduce both first and recurrent stroke. Small clinical studies have shown that statin therapy increases CBF in patients with leukoaraiosis (Ebinger et al., 2012), and improve cerebral vascular function and in patients with lacunar stroke (Pretnar-Oblak et al., 2006). Furthermore, although large clinical trials of statins specifically after lacunar stroke are lacking, the Stroke Prevention by Aggressive Reduction in Cholesterol Levels (SPARCL) trial showed that statin therapy reduces recurrent stroke to a similar degree in small vessel and large artery stroke, but there was a modest but significant increase in hemorrhagic stroke (Amarenco et al., 2009). In contrast, a more recent study found no effect of statin treatment on either incident lacunes or microbleeds (Xiong et al., 2014). Data on the effects of statin treatment on WMH are controversial. For example, the Regression of Cerebral Artery Stenosis (ROCAS) study reported reduced WMH progression (Mok et al., 2009). Similarly, statin use in participants of the VITAmins To Prevent Stroke MRI substudy was found to reduce WMH and decline in executive function (Xiong et al., 2014), whereas no effect was found in a substudy the Prospective Study of Pravastatin in Elderly at Risk (PROSPER; ten Dam et al., 2005).

As with other stroke subtypes, thrombolysis (i.e., using rt-PA) is commonly used for the treatment of patients with cerebral SVD. However, unlike large vessel or cardioembolic stroke, clot formation in lacunar infarction is not considered to be an important mechanism. Nevertheless, some studies have shown that rt-PA improves neurological outcomes of cerebral SVD patients with lacunar infarcts (Mok and Kim, 2015). One concern regarding the use of rt-PA is the possibility of increase risk of hemorrhage in those patients with co-existing WMH or microbleeds (Mok and Kim, 2015). There are similar concerns with the use of anti-platelet drugs for secondary prevention in patients with cerebral SVD (Mok and Kim, 2015).

Potential prophylactic and treatment strategies for arteriopathy in cerebral SVD might include those that target the endothelium of large and small cerebral vessels, as well as the endothelium of the BBB (Bath and Wardlaw, 2015; Poggesi et al., 2015). Given vasoprotective NO bioavailability might be impaired, NO donors may be useful in rescuing the functioning (and potentially the structural abnormalities) of the cerebral vasculature. The clinical efficacy of traditional NO donors (e.g., glyceryl trinitrate) is, however, limited by their susceptibility to tolerance development, decreased effectiveness under oxidative stress and cytotoxic effects (Bullen et al., 2011). Novel NO agents such as nitroxyl (HNO) donors and NO-independent sGC activators (e.g., BAY 58-2667) are proposed to have advantages over these traditional donors (Evgenov et al., 2006; Bullen et al., 2011; Miller et al., 2013). For example, similar to NO, HNO has potent vasodilator properties, but is resistant to both scavenging by superoxide and tolerance development (Bullen et al., 2011; Miller et al., 2013). In addition, it can suppress the activity of Nox2-NADPH oxidase in cerebral vessels and thus may also be effective in ameliorating oxidative stress (Miller et al., 2013). Other potential interventions for improving artery function could include prostacyclin mimics, PPARγ activators, anti-inflammatory agents, phosphodiesterase-5 inhibitors, and Rho-kinase inhibitors (Bath and Wardlaw, 2015).

## Targeting Oxidative Stress as a Therapeutic Strategy?

As discussed in this review, compelling evidence indicates that oxidative stress might be an important contributor to arteriopathy in cerebral SVD (non-amyloid and amyloid), and also the brain injury and cognitive impairment that typically accompanies it. Targeting oxidative stress as a strategy for the treatment of vascular-related diseases has been proposed and discussed by countless researchers over the past decade or so. However, the potential application of this strategy for the prevention and treatment of cerebral SVD is relatively new.

It is important, however, to remember that ROS have important physiological roles including the normal regulation of cerebral vascular function (see Reactive oxygen species and cerebral vascular function). Thus approaches to ameliorate oxidative stress would need to be balanced to ensure that these important physiological roles remain intact.

### Conventional Anti-Oxidants

There has been much debate about the usefulness of bolstering levels of anti-oxidants to ameliorate oxidative stress in vascular disease *per se*. Administration of anti-oxidants such as Vitamins C and E has shown to be beneficial for vascular function in a number of experimental and small clinical trials (Drummond et al., 2011). A small clinical trial showed that Vitamin E supplementation attenuates the development of white matter lesions (Gopalan et al., 2014). Moreover, as discussed, natural anti-oxidants such as Vitamin E and L-carnitine have shown to be beneficial for cognitive impairments found in rodent models of cerebral SVD (Murad et al., 2014; Ueno et al., 2015). However, disappointingly, results of large clinical trials of anti-oxidant supplementation in vascular disease and stroke have largely failed to show any benefit. For example, the Heart Protection Study found no effect of Vitamin C, Vitamin E and β-carotene supplementation on the incidence of vascular events (including stroke) in 20,500 high-risk individuals (Heart Protection Study Collaborative, 2002).

The reasons for the ‘failure’ of such anti-oxidant strategies may relate to the use of suboptimal doses of the vitamins. It is now recognized that supra-physiological concentrations of Vitamins C and E would be required to compete with the reaction of superoxide and NO (Drummond et al., 2011). Also, although the treatment regimes were effective in increasing plasma levels of anti-oxidants, whether this translates to increased levels in the vasculature was not examined. Even if sufficient levels of anti-oxidants were achieved in vascular cells, the anti-oxidants might in fact exert pro-oxidant effects as a result of their conversion to radical species following their reaction with superoxide (Witting et al., 1997, 1999). Either way, the current consensus is that the ‘traditional’ anti-oxidant approach may not be the best strategy for combatting oxidative stress in cerebral SVD (Drummond et al., 2011).

### Synthetic ROS Scavengers

Enhancing the metabolism of superoxide by SOD could be an effective way to ameliorate the impact of this ROS on vascular function. This could be achieved by using native SOD that is modified to include a polyethylene glycol group, which improves its ability to enter cells and stability. The ROS scavenger tempol is cell-permeable and has been used in experimental studies examining the role of oxidative stress in the arteriopathy associated with cerebral SVD risk factors (Kim-Mitsuyama et al., 2005; Mayhan et al., 2008; Modrick et al., 2009; Zhang et al., 2010; Han et al., 2015). Small cell-permeable molecules that mimic native SOD have been developed including, MnTBAP, which have shown some potential therapeutic benefit in experimental studies of cerebral SVD risk factors (Girouard et al., 2006; Mayhan et al., 2008). Further studies are of course necessary to fully establish their real therapeutic efficacy. The therapeutic potential of ebselen (Gpx mimetic, ONOO^-^ scavenger and Nox inhibitor), edavarone (O_2_^-^ scavenger), and the radical trapping agent disufenton sodium (NXY-059), for the treatment of stroke have been tested. Some clinical benefit has been reported for both ebselen and edaravone, however, no benefit has been demonstrated with NXY-059 (Allen and Bayraktutan, 2009).

### Would Inhibition of the NADPH Oxidases be an Effective Strategy?

Evidence supports a role for NADPH oxidase-derived ROS in both arteriopathy and brain damage caused by cerebral SVD and its risk factors. As such, the use of pharmacological inhibitors of the NADPH oxidases may be an effective strategy. Moreover, given research thus far point toward the Nox2-NADPH oxidase isoform as a major contributor, selectively targeting this isoform may be the most effective approach. It is important to remember, however, that Nox2-NADPH oxidase is also expressed in phagocytic immune cells and plays a crucial role in immunological responses. Thus, long-term therapy with inhibitors, which would be necessary for the prevention and treatment of cerebral SVD, is likely to impair immune function and thus could carry significant side effects such as immunosuppression. Over the past decade, numerous inhibitors of the NADPH oxidases have been developed and are well cited in the literature. The mechanism(s) of action and therapeutic potential of many of these inhibitors have been comprehensively reviewed (Miller et al., 2006; Selemidis et al., 2008; Jaquet et al., 2009; Drummond et al., 2011; Altenhofer et al., 2015; Bedard et al., 2015), and therefore will only be reviewed briefly here.

#### Clinically used Drugs That Target NADPH Oxidases and Oxidative Stress

As mentioned, the control of vascular risk factors (e.g., hypertension, hypercholesterolemia, diabetes) is an important primary and secondary preventative strategy for cerebral SVD and associated brain injury/dysfunction. Notably, three of the most effective and frequently prescribed classes of drugs for the treatment of vascular risk factors have been shown to inhibit NADPH oxidases, and thus are likely to mediate their beneficial actions, in part, by reducing oxidative stress. These include the ACE inhibitors, AT_1_ antagonists and the statins. As discussed, angiotensin II acutely and chronically increases the activity of the NADPH oxidases. ACE inhibitors decrease the conversion of angiotensin I to angiotensin II, whereas angiotensin receptor antagonists block receptor mediated effects of angiotensin II. Thus, both of these therapies are likely to reduce ROS levels under conditions of oxidative stress by preventing the activation of the NADPH oxidases by angiotensin II.

Statins are primarily used therapeutically to lower LDL-cholesterol levels, however, it is well documented that statins exert additional cardiovascular benefits. Indeed, statins have been reported to exert direct protective effects on the vasculature by improving endothelial-derived NO bioavailability and thus endothelial function (Erdos et al., 2006; Tanaka et al., 2007; Pretnar-Oblak et al., 2008; Tong et al., 2009). For example, short-term statin treatment improves vasomotor reserve capacity in healthy adults (Sander et al., 2005). Also, statin therapy improves cerebral endothelial function in patients with lacunar stroke, hypertension, and hypercholesterolemia (Pretnar-Oblak et al., 2006, 2008). Moreover, the beneficial effects of statins on CBF and brain injury after cerebral ischemia are dependent on endothelial NO synthase (Endres et al., 1998; Laufs et al., 2000). Evidence suggests that statins augment NO bioavailability, at least in part, via their inhibitory action on RhoA, which negatively regulates eNOS expression (Budzyn et al., 2006). In addition, statins prevent the activation of Rac1, a small G-protein required for NADPH oxidase activation. Consistent with this, statins inhibit cerebral vascular superoxide production in experimental models of diabetes (Erdos et al., 2006), protect against cerebral ischemia, in part, by attenuating oxidative stress (Hayashi et al., 2005), and rescue Aβ-mediated cerebral vascular deficits and oxidative stress in aged APP mice (Tong et al., 2009). Thus, statins may improve cerebral endothelial function by enhancing NO bioavailability and by decreasing oxidative stress.

#### Historical NADPH Oxidase Inhibitors

Diphenyleniodonium and apocynin are the most frequently used NADPH oxidase inhibitors. Indeed, both have been used to demonstrate a role for the NADPH oxidases in the arteriopathy associated with cerebral SVD risk factors and CAA, as well as ischemic brain injury and cognitive impairment. Unfortunately, however, both have non-specific effects that will limit their clinical usefulness. For example, the flavin antagonist, DPI inhibits all flavin-containing enzymes including the NO synthases (Stuehr et al., 1991) and cytochrome P450 enzymes (O’Donnell et al., 1993). Apocynin on the other hand can act as a pro-oxidant under certain conditions and inhibits the formation of prostanoids (Stolk et al., 1994; Vejrazka et al., 2005). Other less commonly used ‘historical’ NAPDH oxidase inhibitors include tosylphenylalanyl chloromethane (TPCK) and aminoethyl benzenesulfonyl fluoride (AEBSF), which inhibit by interfering with the translocation of p47phox and/or p67phox to the Nox catalytic domain. However, both have a number of off-target effects including anti-coagulation (McCarthy and Macey, 1996).

#### Novel Inhibitors

Our growing understanding on the biochemical properties of the NADPH oxidases, as well as how they are activated, has facilitated the identification and development of several novel small-molecule NADPH oxidase inhibitors. The first of these was the peptide gp91ds-tat, which was designed to inhibit the Nox2-NADPH oxidase isoform. It comprises of a conserved sequence of Nox2/gp91phox linked to a 9-amino acid peptide from the HIV coat, which allows the peptide penetrate cells (Rey et al., 2001). Gp91ds-tat has proven to be an invaluable pharmacological tool in studies investigating the role of Nox2-NADPH oxidase in disease. However, being a peptide it is unlikely to have efficacy if administered orally, and thus its therapeutic potential is very limited.

A number of promising NADPH oxidase inhibitors are emerging through high-throughput screening of compound libraries (Altenhofer et al., 2015; **Table 1**). For example, Triazolo pyrimidines (e.g., VAS2870 and VAS3947) were developed in a screening approach for Nox2-NADPH oxidase inhibitors (Stielow et al., 2006; ten Freyhaus et al., 2006). However, it is now apparent that it may also inhibit the activity of Nox4- and Nox5-NADPH oxidases (Kleinschnitz et al., 2010), and thus is not isoform specific. Unfortunately, VAS2870 has poor solubility and off-target effects (e.g., modulating the thiol redox state; Sun et al., 2012), which limits its use *in vivo.* Pyrazolopyridine derivative (e.g., GKT136901 and GKT137831), which are structurally similar to the triazolo pyrimidines, inhibit Nox1-, Nox4-, and Nox5-NADPH oxidases, and are orally bio-available, have a safe profile, and appear to lack significant off target effects (Laleu et al., 2010).

**Table 1:** Novel NADPH oxidase inhibitors: proposed isoform selectivity, mechanism of action, and off-target effects.

Compound	Isoform selectivity	Mechanism of action	Off-target effects	Reference
Gp91ds-tat	Nox2 oxidase	Inhibits assembly of active Nox2 complex, by preventing the interaction of p47phox with Nox2 catalytic subunit.	None reported	Rey et al., 2001; Csanyi et al., 2011
VAS2870	Nox2, Nox4, Nox5 oxidases	Inhibits assembly of active Nox2 complex but not via interfering with the translocation of p47phox. Actions on Nox4 and Nox5 undefined.	Thioalkylates cysteine residues	ten Freyhaus et al., 2006; Kleinschnitz et al., 2010; Sun et al., 2012
Pyrazolopyridine derivative (e.g., GKT136901 and GKT137831)	Nox1, Nox4, Nox5 oxidases	Not defined. May competitively inhibit the substrate NADPH.	None reported	Laleu et al., 2010; Gaggini et al., 2011
S17834	Not defined, although is likely to inhibit at least Nox2, and Nox4 oxidases	Not defined.	Activates AMPK	Cayatte et al., 2001; Zang et al., 2006
Ebselen and analogs (e.g., JM-77b)	Nox1, Nox2, and Nox5 oxidases	Inhibits interaction of p47phox with the membrane bound subunit p22phox	Gpx mimetic; ONOO^-^ scavenger; Moderate eNOS inhibitor	Muller et al., 1984; Zembowicz et al., 1993; Briviba et al., 1996; Bedard and Jaquet, 2012
Fulvene-5	Nox2, Nox4 oxidases	Not defined	Not determined	Bhandarkar et al., 2009
Triphenylmethane derivatives (e.g., Brilliant green, Gentian violet),	Nox2, Nox4 oxidases	Not defined. May interfere with extracellular Nox domains. Similar structure to the flavin antagonist, DPI.	Not determined	Perry et al., 2006; Munson et al., 2012
Grindelic acid	Nox4 oxidase	Not defined	May inhibit other flavoproteins	Kofler et al., 2013
ML171 (2-acetylphenothiazine)	Nox1 > Nox2, and Nox4 oxidases	Not defined	May inhibit serotonin and adrenergic receptors	Gianni et al., 2010

*AMPK, adenosine monophosphate-activated protein kinase (AMPK); DPI, diphenyleneiodonium; eNOS, endothelial nitric oxide synthase; Gpx, glutathione peroxidase; ONOO^-^, peroxynitritre.*

The flavonoid derivative S17834 was proposed to be a NADPH oxidase inhibitor based on its ability to inhibit superoxide production by cultured human umbilical vein endothelial cells (HUVEC) and in isolated endothelial cell membranes, which express Nox2- and/or Nox4-NADPH oxidase isoforms (Cayatte et al., 2001). Ebselen and several of its selenium-containing analogs have been identified as inhibitors of Nox2 and Nox1 oxidase, with one analog (JM-77b) exhibited potential Nox2 selectivity. Notably, the Nox inhibitory effect of ebselen occurred at concentrations well below those at which it functions as a Gpx mimetic, which would allow it to be used as ‘specific Nox inhibitor’ (Muller et al., 1984). However, the Gpx properties (and ONOO^-^ scavenging properties) of ebselen clearly could be considered advantageous in the setting of oxidative stress. Another potential advantage of ebselen is that it orally available and appears to have a safe profile (Yamaguchi et al., 1998). Other promising NADPH oxidase inhibitors include fulvene-5, triphenylmethane derivatives (e.g., Brilliant green), grindelic acid, and ML171 (also known as 2-acetylphenothiazine; recently reviewed in (Altenhofer et al., 2015; **Table 1**).

It is clear therefore that although significant progress has been made over the past decade or so, more research is needed to identify fully isoform selective Nox inhibitors with favorable bioavailability. Further insight into the molecular structure of the Nox catalytic subunits, their interaction with other NADPH oxidase subunits, and how the Nox’s differ from each other, is pivotal for the development of the next generation of selective inhibitors.

## Conclusion and Perspectives

**FIGURE 1:**
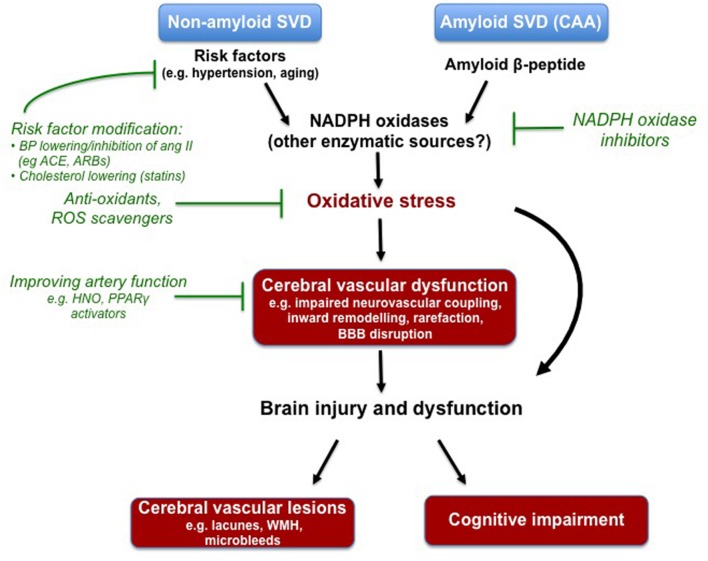
**Compelling experimental evidence suggests that oxidative stress underlies some aspects of cerebral vascular dysfunction associated with known risk factors for non-amyloid cerebral small vessel disease (SVD), and contributes to the deleterious actions of soluble and deposited amyloid-beta in the setting of cerebral amyloid angiopathy (CAA).** The NADPH oxidases appear to be a major driver of this oxidative stress, however, future research is needed to elucidate the potential contributes of other enzymes. Various experimental models have been employed to study the cerebral vascular lesions and cognitive impairment that typically manifest in cerebral SVD. Oxidative stress and the NADPH oxidases are once again implicated. Despite evidence of its devastating effects on the brain, there are at currently no specific pharmacological strategies to prevent or treat cerebral SVD. Modifying risk factors with drugs already in the clinic (e.g., ACE inhibitors, angiotensin II receptor blockers) seems like a logical approach. Moreover, agents that target the endothelium (i.e., increasing nitric oxide bioavailability) may be useful in rescuing the functioning of the cerebral vasculature. Lastly, strategies to combat oxidative stress (e.g., NADPH oxidase inhibitors) may also be a novel strategy.

From the evidence outlined in this review, it is apparent that oxidative stress may be a contributor to some of the features of arteriopathy associated with both non-amyloid and amyloid forms of cerebral SVD, and its risk factors (**Figure 1**). Moreover, there is ample evidence of its potential contribution to ischemia-induced brain injury and cognitive impairment, important manifestations of cerebral SVD. Compelling evidence points toward the Nox2-NADPH oxidase isoforms, as a key mediator of oxidative damage in the brain and its circulation. However, future work is needed to evaluate the contribution of other NADPH oxidase isoforms (e.g., Nox1- and Nox4-containing isoforms), as well as the importance of other sources of ROS such as the mitochondria, COX and uncoupled eNOS. This latter point notwithstanding, ROS are clearly compelling targets for the prevention and treatment of cerebral SVD. Moreover, the concept of inhibiting the production of ROS, by the NADPH oxidases for example, should offer advantages of conventional anti-oxidants. The NADPH oxidases have been linked to numerous vascular-related diseases and as such the design of novel selective NADPH oxidase inhibitors is currently an area of intense research focus. However, a major challenge in the development of such agents will be the retainment of at least some NADPH oxidase activity for normal immunological defense and for the physiological regulation of cerebral vascular function. Lastly, it is important to acknowledge that the majority of the evidence discussed in this review comes from experimental models of cerebral SVD and risk factors. As such, an essential goal of future work will be to elucidate the extent to which these experimental findings translate to the human scenario, and whether therapeutic approaches to alleviate oxidative stress provide real benefits with respect to reducing cerebral vascular events and cognitive impairment.

## Author Contributions

Both AM and TD conceived, planned, wrote, and edited this review article.

## Conflict of Interest Statement

The authors declare that the research was conducted in the absence of any commercial or financial relationships that could be construed as a potential conflict of interest. The Associate Editor CS declares that, despite being affiliated to the same institution as the author TD, the review process was handled objectively and no conflict of interest exists.
